# B cell mechanosensing regulates ER remodeling at the immune synapse

**DOI:** 10.3389/fimmu.2024.1464000

**Published:** 2024-10-07

**Authors:** Isidora Riobó, María Isabel Yuseff

**Affiliations:** Immune Cell Biology Lab, Pontificia Universidad Católica de Chile, Facultad de Ciencias Biológicas, Santiago, Chile

**Keywords:** B-cell, immune synapse, endoplasmic reticulum, cytoskeleton rearrangements, mechanotransduction

## Abstract

**Introduction:**

Engagement of the B-cell receptor with immobilized antigens triggers the formation of an immune synapse (IS), a complex cellular platform where B-cells recruit signaling molecules and reposition lysosomes to promote antigen uptake and processing. Calcium efflux from the endoplasmic reticulum (ER) released upon BCR stimulation is necessary to promote B-cell survival and differentiation. Whether the spatial organization of the ER within the B-cell synapse can tune IS function and B-cell activation remains unaddressed. Here, we characterized ER structure and interaction with the microtubule network during BCR activation and evaluated how mechanical cues arising from antigen presenting surfaces affect this process.

**Methods:**

B-cells were cultured on surfaces of varying stiffness coated with BCR ligands, fixed, and stained for the ER and microtubule network. Imaging analysis was used to assess the distribution of the ER and microtubules at the IS.

**Results:**

Upon BCR activation, the ER is redistributed towards the IS independently of peripheral microtubules and accumulates around the microtubule-organization center. Furthermore, this remodeling is also dependent on substrate stiffness, where greater stiffness triggers enhanced redistribution of the ER.

**Discussion:**

Our results highlight how spatial reorganization of the ER is coupled to the context of antigen recognition and could tune B-cell responses. Additionally, we provide novel evidence that the structural maturation of the ER in plasma cells is initiated during early activation of B-cells.

## 1 Introduction

B-cells are part of the adaptive immune system that upon activation can differentiate into memory B-cells or antigen-specific antibody-secretory plasma cells. B-cell activation begins when the BCR recognizes a cognate antigen usually tethered to an antigen presenting cell (APC), triggering the formation of an immune synapse (IS) (1). This structure is characterized by lysosome polarization towards the site of contact with the antigen in addition to intense cytoskeleton remodeling, favoring the formation of actin foci and cell spreading (2–4). These internal rearrangements enable the release of lysosomal content into the extracellular environment, facilitating antigen extraction. Uptaken antigens are further processed into peptides, which are mounted on molecules of the major histocompatibility complex II (MHC-II) to be presented on the membrane for T-cell recognition. Finally, cytokines released from these cells promote B-cell differentiation into plasma, memory, or germinal center-forming cells (5, 6).

B-cells can recognize antigens either in a soluble form (~0.01 kPa) or immobilized on different surfaces, such as the membrane of antigen presenting cells (APC) or tethered to the extracellular matrix (~20 kPa), or viral capsids (~100 MPa) (7–9). Additionally, APCs exhibit different surface stiffnesses, which generally increases under inflammatory environments (7) and thus B-cells are exposed to antigens associated to diverse physical properties under different conditions. Integrins expressed by B-cells such as LFA-1, along with the BCR, are mechanosensory; that is, they can sense the stiffness of the substrate that anchors the antigen, modulating B-cell activation. Antigens tethered to substrates that display higher stiffness trigger increased BCR downstream signaling, leading to augmented cytoskeleton rearrangements and spreading responses (10). While its effect on the efficiency of IS formation and consequently the extraction and presentation of antigens are not known, it has been established that the stiffness of the surface where antigens are encountered determines the extraction method utilized by the B-cells (11, 12). Antigens anchored on soft, flexible surfaces are uptaken mechanically by B-cells, where pulling forces mediated by myosin-IIA enable the internalization of BCR-antigen complexes (11, 13). On the other hand, when antigens are tethered to a stiffer surface, B-cells reorientate their MTOC to direct the transport of lysosomes to the IS where their content is released to favor proteolytic extraction (14, 15).

Activation of B-cells also leads to calcium release from the ER and is associated to the transcription of differentiation genes (16). Despite the diverse functions and dynamic nature of the ER, no other role or structural changes during IS formation have been described for this organelle in B-cells. However, several functions of the ER, described in other cells, could be used by B-cells to promote IS formation and activation. For instance, epithelial cells require ER-exit site (ERES) formation to adapt to mechanical stress, however the formation of ERES in B-cells during IS formation and in response to mechanical cues has not been evaluated. On the other hand, ER tubules mediate membrane contact sites with other organelles for lipid and calcium transfer in addition to fission and fusion regulation (17, 18).

Considering the dynamic structure of the ER and its diverse functions, we investigated ER structural changes triggered by BCR activation. Our findings elucidate for the first time how B-cells reshape their ER upon antigen-stimulation in a substrate stiffness-dependent manner and opens new opportunities to evaluate possible functional implications in B-cell activation and differentiation to plasma cells.

## 2 Materials and methods

### 2.1 Cell culture and mice

Mouse lymphoma A20 cell line was cultured in CLICK medium (RPMI 1640 with glutamax, 0.1% β-mercaptoethanol, 100U/mL penicillin, 100μg/mL streptomycin and 1mM sodium pyruvate) supplemented with 10% heat inactivated fetal bovine serum (FBS). Primary B-cells were purified from 2 week old C57BL/6 mice using a B cell Isolation Kit (Miltenyi Biotec).

### 2.2 Antibodies

The following primary antibodies were used: rat anti-mouse α-tubulin (MERCK #MAB1864), rabbit anti-mouse KTN1 (Proteintech #19841) and rabbit anti-mouse calnexin (Abcam #ab22595). Secondary antibodies: donkey anti-rat Alexa Fluor™647/488 (Jackson Immunoresearch #712606153/#712546150), donkey anti-rabbit Alexa Fluor™488/Cy3 (Jackson Immunoresearch #711546152/#711166152), phalloidin Alexa Fluor™647 (Invitrogen #A2287) and phalloidin Alexa Fluor™ Plus 405 (Invitrogen #A30104).

### 2.3 Cell transfection and electroporation

Transfection with VapA-mCherry and mCherry was performed by electroporation with Nucleofactor R T16 kit (Lonza, Gaithersburg, MD) using 6μg of plasmid per 5x10^6^ cells. Cells were used after 18 ± 2 hours in culture at 37°C and 5% CO_2_ in CLICK medium (RPMI 1640, 10% fetal bovine serum, 1% penicillin–streptomycin, 0.1% β-mercaptoethanol, and 2% sodium pyruvate).

### 2.4 Glass coverslips and antigen-coated beads preparation for B-cell activation

Glass coverslips of 10mm diameter were coated using 0.13μg/μL BCR^+^-ligand (F(ab′)2 goat anti-mouse IgG) (Jackson ImmunoResearch, 115-006-146) in PBS; 40µL drops were seeded on Parafilm™, and coverslips were placed on top. They were incubated at 4°C overnight and washed 3 times with PBS before use. poly-L-lysine, was used as a non-activating condition.

For B-cell–bead conjugate formation, we used the protocol described in 19, 4×10^7^ latex NH_2_ beads (Poly-science) were activated with 8% glutaraldehyde (Merck) for 2h at RT. Beads were then washed twice with PBS and incubated overnight with 20μg of different ligands (F(ab′)2 goat anti-mouse-IgG, or F(ab′)2 goat anti-mouse-IgM or BSA, MP Biomedicals). Beads were blocked and washed with PBS.

### 2.5 Tunable-stiffness polyacrylamide gels preparation

18mm diameter glass coverslips were activated with a mix of 2% 3-Aminopropyltrimethoxysilane (3-APTMS) (Sigma Aldrich 13822-56-5) and 2% glacial acetic acid in 96% ethanol for 3 minutes, washed with 70% ethanol and dried with lens cleaning paper. Another set of 12mm coverslips were waterproofed using Rain-X™ for 3 minutes and then washed with pure water. Soft PAA gels of 0.3kPa were prepared using 30μl of 2% bis-acrylamide solution (Bio-Rad #1610142), 75μl of 40% acrylamide (Bio-Rad #1610140), 10μl of 10% APS, 1 μL of TEMED and 895 μL of PBS. Stiff PAA gels of 13kPa were prepared with 125μl of 2% bis-acrylamide solution (Bio-Rad #1610142), 234μl of 40% acrylamide (Bio-Rad #1610140), 6.25μl of 10% APS, 1.875μl of TEMED and 883 μL of PBS.

A 10μL drop of the PAA gel mix was placed onto the activated side of a 18mm coverslips, and covered with a treated 12mm coverslip, waterproofed side towards the drop, making a “sandwich” with the PAA gel mix in the middle. After 30 min for polymerization to take place, the top coverslip was gently removed and the gels were washed with PBS 1X. PAA gels were stored covered in PBS at 4°C overnight and used within 48 hours.

For conjugation with BCR^+^-ligand, PAA gels were treated with 10mM Pierce™ Sulfo-Sanpah (Thermo Scientific #A35395) in PBS and exposed to 365nm UV light for 10 min at room temperature and washed 3 times with PBS. Finally, slides were incubated with BCR^+^-ligand similarly to glass coverslips.

### 2.6 Activation of B-cells for immunofluorescence

B-cells were resuspended in 100 μL of CLICK medium + 5% FBS and the following amounts seeded: For glass coverslips, 200.000 cells for BCR^–^ligand coverslips and 80.000 cells for BCR^+^-ligand coverslips; for PAA gels, 250.000 cells for non-conjugated gels and 100.000 cells for conjugated ones. They were incubated for different time points at 37°C and 5% CO_2_. After each time point, the medium was discarded and the cells washed with cold PBS and fixed with 3% PFA, 40 μL (glass) or 60 μL (PAA gel), for 10 minutes at RT. Cells were washed three more times with cold PBS and blocked with the same volumes previously mentioned for glass and PAA gels using blocking buffer (2% BSA and 0.3M glycine in PBS).

For bead activation, B-cells were mixed with beads in a 1:3 ratio for 0 and 10 minutes, and 1:1 ratio for 60 min activation time points. The mix was then seeded on poly-L-lysine coated coverslips.

Primary antibodies were prepared in permeabilization buffer (0.2% BSA and 0.05% saponin in PBS) and 60 μL were added on top of the PAA gels. For glass coverslips, 40 μL of the antibody solution was placed on a parafilm and the coverslip placed on top, with the side of the cells towards the drop. Cells were incubated in a humid chamber at 4°C ON. After addition of the secondary antibody, cells were incubated at RT for 1h in the dark. Coverslips and PAA gels were then washed three times with permeabilization buffer for 5 minutes and once with PBS. After removal of the PBS they were mounted using 4 μL (glass) or 6 μL (PAA gel) of Fluoromont-G™.

### 2.7 Nocodazole treatment

Before activation, cells were treated with 10μM nocodazole for 1h, without removing the drug before incubation on glass coverslips.

### 2.8 Cell imaging

Confocal images were acquired using a Zeiss LSM 880 Airyscan confocal microscope with 63X/1.4 immersion objective and Inverted Nikon Ti2-E with 100X/1.45 immersion objective. All Z-stacks were obtained with 0.14 μm between slices. Epifluorescence images were acquired using a Nikon Ti Eclipse epifluorescence microscope with an X60/1.25NA objective.

### 2.9 Image analysis

Image processing and analysis were performed using the Fiji software, based on ImageJ, and R software. GraphPad Prism 9 was used for statistical analysis.

#### 2.9.1 Z scan

For fluorescence analysis in each cell slice we used a proprietary macros and script made for Fiji (ImageJ) and R, respectively (Available and explained in detail at https://github.com/YuseffLab/Z-stack-multimeasure). Broadly, we measured the raw fluorescence for each slice of an image. Then the percentage of each slice was calculated with respect to the whole cell fluorescence (sum of slices). For quantification of fluorescence at the IS, we considered the sum of the first 10% of slices for each cell.

#### 2.9.2 Bead polarization analysis

Polarization index quantification was performed as described in 19 using a macro for Fiji software.

#### 2.9.3 Colocalization

For colocalization analysis the Coloc2 Fiji (ImageJ) plugin was used. Colocalization was performed on the first 10% of the total slices using the cell area as the ROI. Pearson’s Coefficient parameter was used for statistical analysis.

#### 2.9.4 Density around the MTOC

To quantify the density of a signal around the MTOC, we used the Z-slice where the MTOC label was found, along with the slices directly before and after it. A circle (Diameter: 4μm for A20 and 2μm for primary cells) was centered at the MTOC and the mean fluorescence of the signal was calculated. Line scans were performed using a line across the cell width or the MTOC and the “Plot Profile” function of Fiji (ImageJ).

#### 2.9.5 Area and volume quantification

Area and volume of the ER were quantified using Fiji (ImageJ). For volume quantification we used the function “3D Objects Counter”. 3D reconstructions were made with the plugin “3D Viewer”.

## 3 Results

### 3.1 ER localizes to the immune synapse of activated B-cells

**Figure 1:**
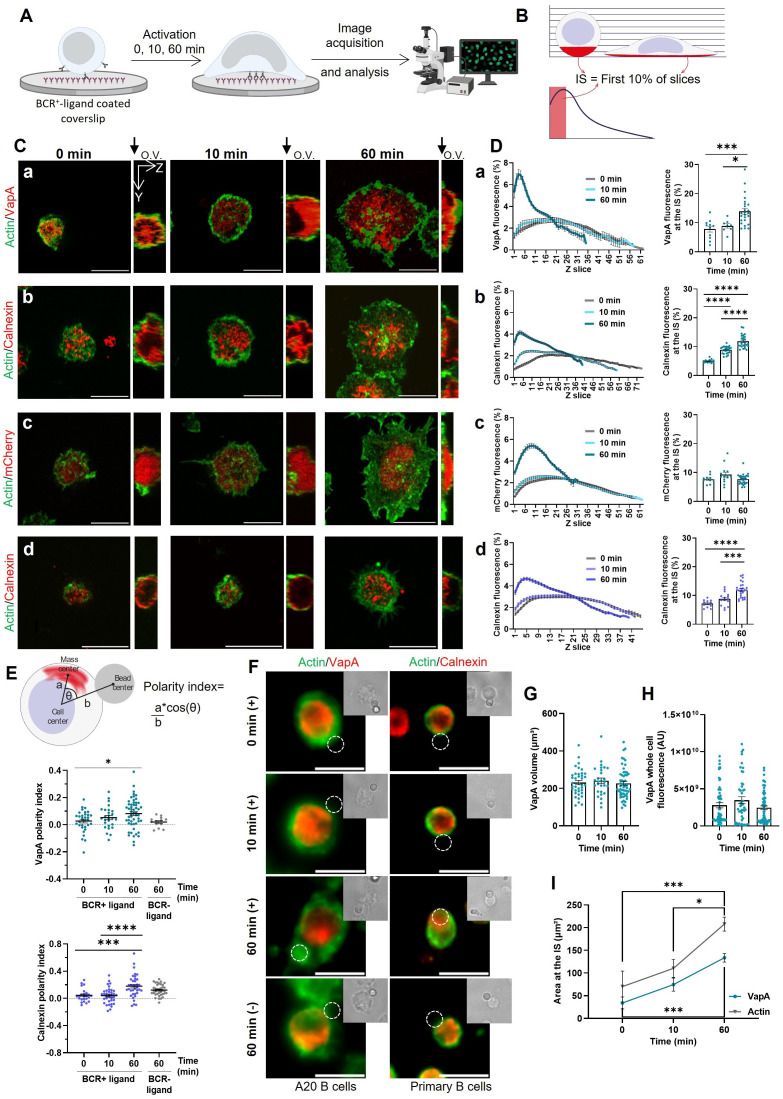
Reorganization of the endoplasmic reticulum at the IS. **(A)** B-cells were activated on glass coverslips covered with BCR^+^-ligand for different time points, fixed and then used for immunofluorescence imaging. **(B)** Z scan analysis representation. The fluorescence for every slice of the Z stack was quantified and the first 10% of each cell was quantified in order to compare similar volumes. **(C)** Representative confocal images of A20 B-cells expressing VapA-mCherry (a), mCherry (c) or stained for calnexin (b), and primary B-cells stained for calnexin (d) activated for different time points. Orthogonal view (O.V.) is shown, where the glass is located at the left. **(D)** Left: Z scan profile of B-cells activated for different time points. Right: Signal fluorescence at the IS per time point. **(E)** Top: Scheme depicting polarity index measurement. Bottom: Polarity index quantification for A20 and primary B-cells. **(F)** Representative images of A20 and primary B-cells activated with beads coated with BCR+-ligand or BCR^–^ligand. **(G)** VapA volume quantification per time point. **(H)** VapA whole cell fluorescence per time point. **(I)** VapA and actin area at the IS. Scale bar 10 μm. Error bars represent standard media error. ANOVA followed by Tukey multiple comparisons. *p<0.05, ***p<0.001, ****p<0.0001.

The ER is a highly dynamic organelle formed by sheets and tubules, which are constantly being reshaped by multiple remodeling proteins (20, 21). To assess whether BCR activation is associated to ER remodeling, we first evaluated changes in the distribution of ER membranes at the immune synapse. To this end, B-cells were activated using glass coverslips coated with BCR^+^-ligand (F(ab′)2 anti-IgG) and stained for actin to simultaneously evaluate cell spreading (**Figure 1A**). VapA is an ER membrane protein that participates in multiple membrane contacts sites (20). Using B-cells expressing VapA-mCherry, its enrichment at the IS was assessed considering the plane of the synapse as the first 10% of the Z-slices that were imaged closest to the glass, so that a similar volume is considered in all activation time points (**Figure 1B**). After 60 min of activation an increase of the VapA signal was observed at the IS (**Figures 1C, D**). Importantly, similar results were obtained when labeling the ER with an antibody against calnexin, indicating that the accumulation of ER markers at the IS, did not merely result from over expression of VapA (**Figures 1C, D**). This was further confirmed by showing that, expression mCherry alone did not re-distribute to the IS of B cells upon activation (**Figures 1C, D**). We next asked whether ER redistribution to the IS also occurred in primary B-cells. To this end, primary B cells isolated from mouse spleen were activated on coverslips coated with BCR^+^-ligands (F(ab′)2 anti-IgM) and stained for calnexin. Indeed, equivalent results were obtained, where B cells showed an increased signal of the ER at the IS upon BCR activation (**Figures 1C, D**). Finally, to further evaluate polarization of the ER towards the IS, A20 and primary B-cells were activated using F(ab′)2 anti-IgG or anti-IgM coated beads respectively. After different activation time points, cells were fixed and stained for calnexin and polarization of the ER was calculated as illustrated in **Figure 1E**. In both cases a significant polarization of the ER was observed upon BCR stimulation (**Figures 1E, F**). This was not observed in cells incubated with non-activating beads (anti-IgM and BSA coated beads for A20 and primary B-cells, respectively), indicating that it relied on BCR activation.

To determine whether the increase of ER at the synaptic plane was due to a net growth of the organelle or a dynamic equilibrium between the formation and breakdown of its membranes, the volume of VapA was quantified. No differences in ER volume were observed after 0, 10 and 60 min of activation (**Figure 1G**). Additionally, no differences were observed in the fluorescence signal of VapA in the entire cell upon activation (**Figure 1H**). Interestingly, an increase in XY extension of the ER at the synaptic plane was observed (**Figure 1I**, colored line) and correlated with the spreading area of the cell in a time dependent manner (**Figure 1I**, gray line).

### 3.2 ER-cytoskeleton interactions change upon BCR activation

**Figure 2:**
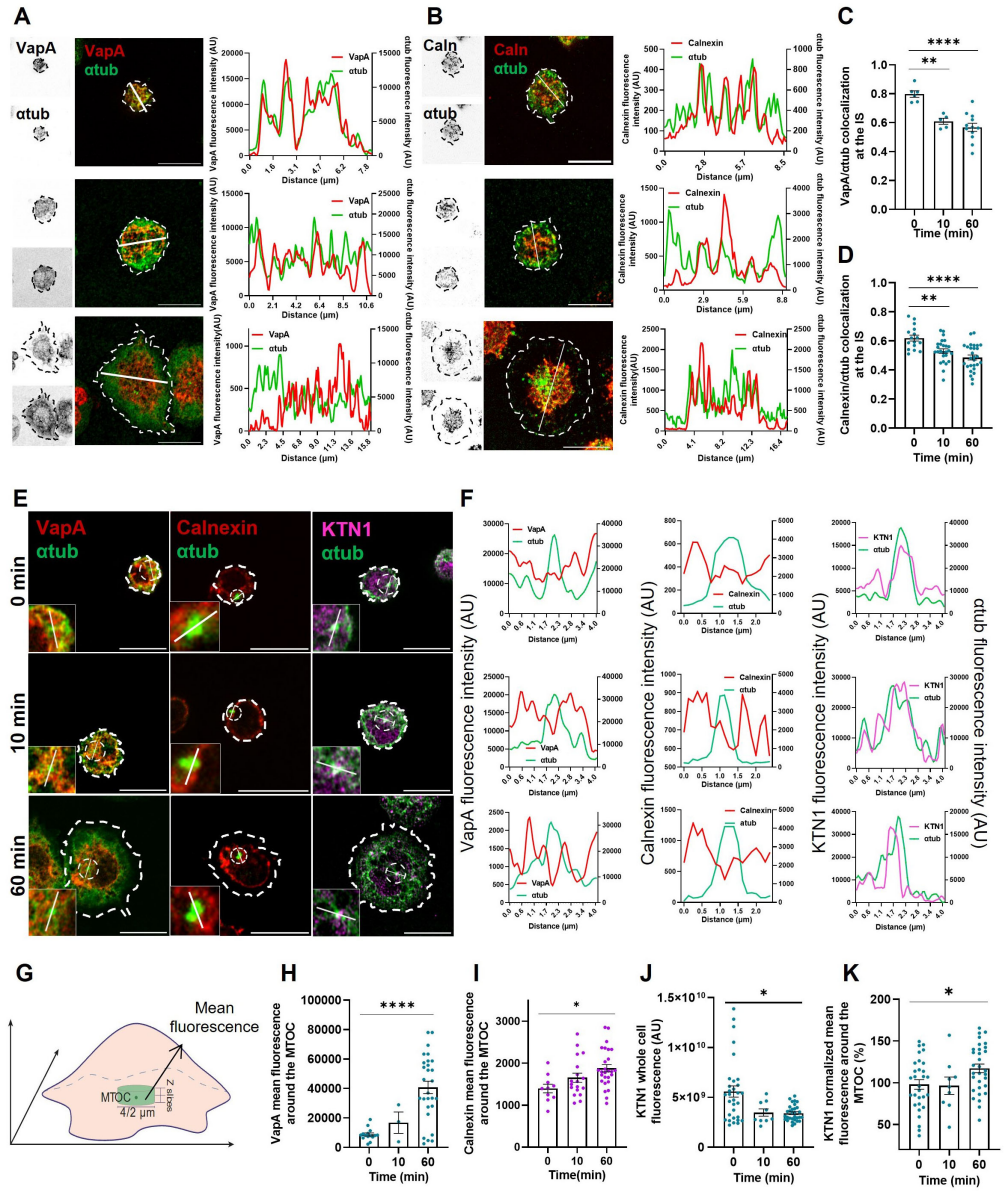
Endoplasmic reticulum interactions with microtubule cytoskeleton. **(A, B)** Left: Representative confocal images of A20 B-cells expressing VapA-mCherry or stained for calnexin activated for different time points. Right: Fluorescence intensity distribution of α-tubulin and VapA/calnexin across the cell (white line). **(C, D)** Colocalization test for VapA/calnexin and α-tubulin. First 10% of slices for each image was used. **(E)** Confocal images of the best slice for MTOC visualization of B-cells activated for different time points. The inset shows MTOC localization and dashed circle shows area of analysis for mean around MTOC. **(F)** Fluorescence intensity distribution of α-tubulin and VapA (a), calnexin (primary B cells) (b) and KTN1 (c) across the MTOC. **(G)** Schematics depicting analysis for mean fluorescence around the MTOC. A 4μm (A20 cell line) or 2 μm (primary B-cells) diameter circle was used to measure the mean fluorescence at the MTOC slice, in addition to one upper and one lower slice. **(H, I, K)** VapA **(H)**, calnexin **(I)** and KTN1 **(K)** mean fluorescence around the MTOC. **(J)** KTN1 whole cell fluorescence per time point. ANOVA followed by Tukey multiple comparisons. *p<0.05, **p<0.01, ****p<0.0001.

Our observations suggest that BCR activation triggers a redistribution of the ER throughout the whole cell. The growth of ER tubules is guided by microtubules through mechanisms that are regulated by different cues, such as remodeling proteins and posttranslational modifications of microtubules (22–24). Thus, we sought to evaluate ER-microtubule interactions during IS formation. To this end, colocalization between VapA and α-tubulin was quantified using Pearson’s coefficient in B-cells expressing VapA-mCherry activated on coverslips and stained for α-tubulin as previously described. A significant decrease in VapA/α-tubulin colocalization was observed after 10 min of B-cell activation (**Figures 2A, C**), suggesting that interaction sites between ER and microtubules decrease upon BCR activation. No differences in transfection levels of VapA were observed at different time points that could affect the degree of colocalization with microtubules (**Figure 1H**). To formally rule out this possibility, we labeled the ER with calnexin and evaluated its colocalization with microtubules, which revealed similar results confirming that BCR activation leads to a decrease in ER-microtubules interactions (**Figures 2B, D**).

It has been well described that the MTOC has a key role in orchestrating cell polarity during IS formation in B-cells (14, 15, 25). Therefore, to further study ER-microtubule interactions, we next evaluated whether dynamic changes in ER membranes occur at this site. For this purpose, VapA fluorescence around the MTOC was quantified in resting and BCR-stimulated B-cells as depicted in **Figure 2G**. Although the ER localizes around the MTOC at all activation times (**Figures 2E, F**), our results revealed that after 1h of stimulation the association of ER to this region of the cell increased significantly (**Figure 2H**). Similar results were obtained with primary B-cells (**Figures 2E, F, I**).

Interestingly, ER-remodeling proteins that interact with microtubules display different localization according to the intracellular region. One such protein is kinectin 1 (KTN1), which localizes to the perinuclear zone (24), and has two roles; it promotes the formation of ER sheets, maintaining their flatness and can interact with kinesin to promote the growth of ER tubules (20). Given this background, we aimed to evaluate the dynamics of KTN1 in B-cell activation and its association to the ER. Our results show that KTN1 is found closer to the MTOC than VapA (**Figures 2E, F**). Interestingly, BCR activation triggered the accumulation of KTN1 around the MTOC, despite of the reduction observed in total levels (**Figures 2J, K**). These results suggest a possible role of KTN1 in ER association to the MTOC, where it concentrates in response to BCR activation.

### 3.3 ER membrane recruitment at the IS does not depend on microtubules

**Figure 3:**
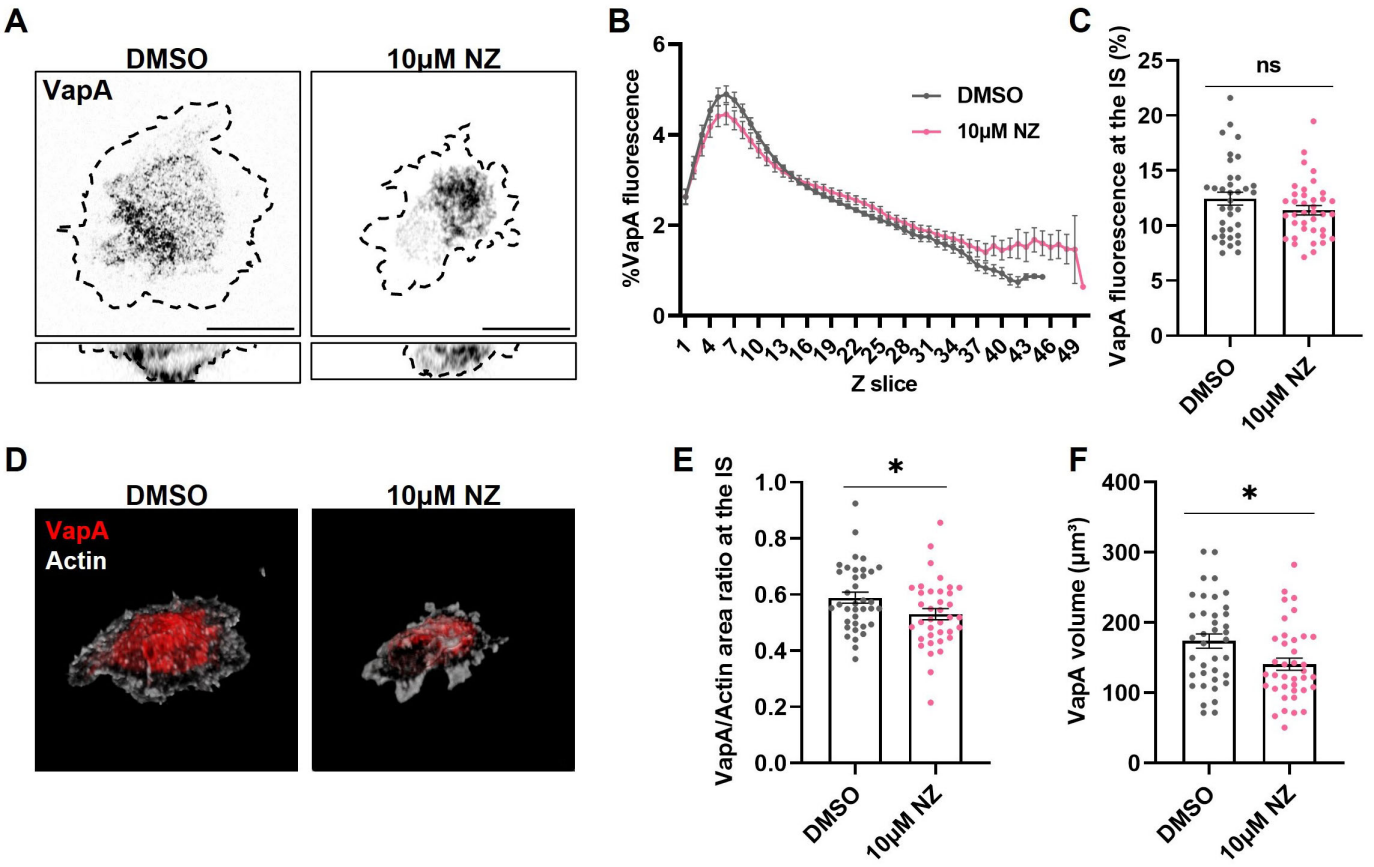
Effect of microtubule disruption on endoplasmic reticulum remodeling. **(A)** Representative confocal images of B-cells activated for 1h under different conditions. Discontinuous border represents actin. Scale bar 10 μm. **(B, C)** VapA Z scan profile of B-cells activated on different conditions and its fluorescence at the IS for images on **(A)**. **(D)** B-cells 3D representation of actin (white) and VapA (red). **(E, F)** VapA area and volume quantification for images on **(A)**. Error bars represent standard media error. Student’s t-test. *p<0.05.

Regarding the decrease in colocalization of ER-microtubules upon B cell stimulation, we next studied the importance of these interactions for ER remodeling during IS formation. For this purpose, B-cells expressing VapA-mCherry were treated with 10μM nocodazole, a concentration previously shown to impair microtubule polymerization in these cells (14), activated on coverslips and stained for α-tubulin as described above. The treatment did not disrupt ER distribution at the synaptic plane, as treated cells also showed increased VapA fluorescence in the slices closest to the coverslip (**Figures 3A–C**). However, considering the relevance of the microtubule network for ER growth, we then evaluated how treatment affects the total extension of the label of VapA. To this end, the area corresponding to VapA at the XY plane of the IS was measured, and then divided by the spreading area of the cell delimited by cortical actin. Nocodazole-treated cells showed less extension of VapA after 1h of activation (**Figures 3A, E**), affecting ER spreading upon BCR activation, as well as a decrease of VapA volume (**Figures 3D, F**). Nocodazole-treated cells displayed a smaller ER which, upon BCR activation, localized at the center of the synapse plane. Altogether, these results suggest that disrupting the dynamics of the microtubule network affects the growth of the ER, but not its redistribution towards the IS.

### 3.4 Substrate stiffness regulates ER distribution in B cells

So far, our results indicate that activation of B-cells seeded on glass causes the ER to (1) increase its distribution at the IS, (2) decrease its colocalization with microtubules, (3) increase its density around the MTOC and (4) extend its tubules towards the cell border.

The BCR harbors mechanosensitive capacity and thus the physical properties of the substrate to which the antigen is anchored can influence its extraction (11, 12). Interestingly, mechanical strains were shown to increase ER-exit sites (ERES) (26), demonstrating the ability of this organelle to respond to mechanical stimuli.

**Figure 4:**
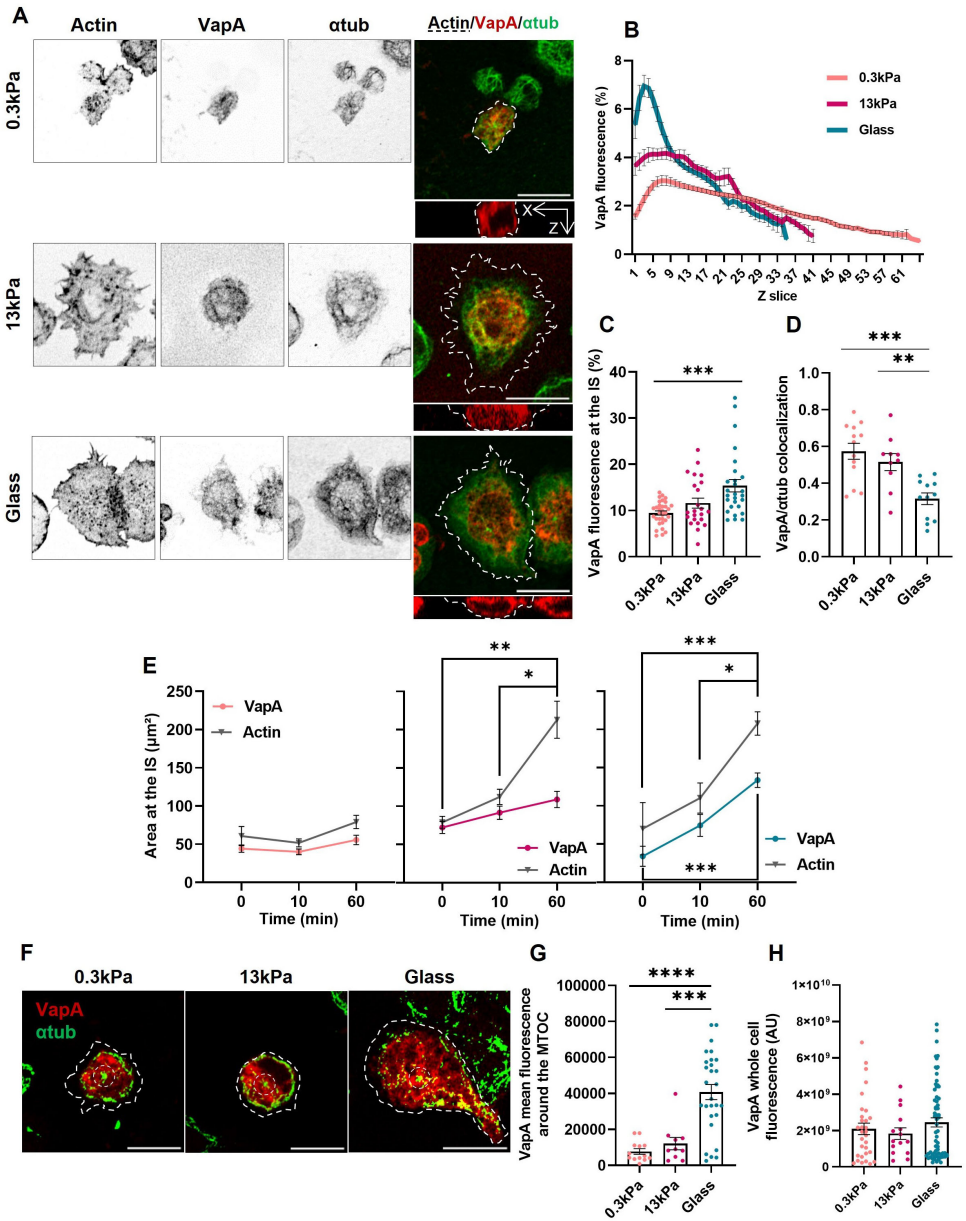
Regulation of endoplasmic reticulum remodeling by substrate stiffness. **(A)** Representative confocal images of B-cells activated for 1h on substrates of different rigidities (Cell shown for glass conditions is the same as **Figure 2A**). **(B, C)** VapA Z scan profile of B-cells activated on different substrates and its fluorescence at the IS. **(D)** Colocalization of VapA and α-tubulin. First 10% of slices for each image was used. **(E)** VapA and actin cell spreading area of B-cells activated on different substrates. **(F)** Representative confocal images of B-cells activated for 1h on different substrates. α-tubulin contrast was augmented for better MTOC visualization. Discontinuous borders represent actin. **(G)** VapA mean fluorescence around the MTOC. **(H)** VapA whole cell fluorescence of B-cells seeded on different conditions for 1 hour. Scale bar 10 μm. Error bars represent standard media error. ANOVA followed by Tukey multiple comparisons. *p<0.05, **p<0.01, ***p<0.001, ****p<0.001.

To assess whether external physical cues alter the ER dynamics, B-cells expressing VapA-mCherry were seeded on 0.3 and 13kPa PAA gels, which mimic physiological and pathological mechanical cues encountered by B-cells. Next, the morphology of the ER at the IS was evaluated using similar analyses as described above. Our results show that activation of B-cells on 0.3kPa and 13kPa gels showed less recruitment of ER membranes to the IS compared to glass activated cells (**Figures 4A–C**), evidenced by a more even distribution of VapA fluorescence throughout the cell height (**Figure 4B**), and in diminished accumulation in the first 10% of the Z-slices (**Figure 4C**). Additionally, B-cells activated on softer substrates failed to trigger decolocalization between the ER and microtubules (**Figures 4A, D**), suggesting that this process depended on mechanosensing by B-cells. Next, we further evaluated ER extension together with cell spreading in B-cells activated on glass, and 13kPa and 0.3kPa gels. Our results show that B-cells activated on PAA gels do not show significant increase of ER area at the IS, compared to B-cells seeded on glass, which displayed higher ER areas at the synaptic plane (**Figure 4E**, colored lines). Furthermore, this enlargement occurs concurrently with the increased in actin spreading (**Figure 4E**, gray lines). On the other hand, B-cells activated at 0.3kPa showed neither actin nor ER spreading.

Finally, when measuring VapA fluorescence around the MTOC, cells activated on glass, unlike those activated on gels, accumulated higher levels of ER at the MTOC, supporting a role for substrate stiffness as a cue in ER organization (**Figures 4F, G**). Once again, the transfection levels of VapA remain consistent across the different conditions, ensuring they do not affect the results obtained (**Figure 4H**). In conclusion, activation of B-cells on stiffer substrates leads to greater changes in ER remodeling and its interactions with microtubules.

## 4 Discussion

The formation of an IS is intimately associated to changes in B-cell architecture. Research on B-cells has extensively covered cytoskeleton remodeling and dynamics of organelles including lysosomes and the nucleus during activation. However, the remodeling of the ER at the IS has not been explored until now, despite being the largest organelle in the cell and fulfilling multiple functions that could enhance B-cell activation, in addition to its role as a calcium reservoir.

Our results show that BCR activation triggers ER redistribution throughout the whole cell, increasing its surface at the IS without involving a net growth in volume, suggesting that there is a balance between gain and loss of ER membrane. The ER reaches the synaptic plane independently of microtubule polymerization. However, disruption of microtubule dynamics leads to a reduced ER volume, suggesting that they are crucial to maintain the balance between ER membrane gain and loss.

BCR activation triggered the uncoupling between ER and microtubules and thus increased in ER growth might be mediated by complexes such as the tip attachment complex (TAC), which consists of STIM1 on the ER and EB1 on the microtubule tip, which does not involve contact points along ER and microtubules (20). STIM1 is activated upon BCR activation downstream of calcium release from the ER and binds to Orai1 calcium channels in the plasma membrane, allowing store-operated calcium entry (SOCE), activating signaling cascades and transcription factors required for B-cell differentiation (16). Whether STIM1 activation is related to an increase in TAC-mediated growth is not known, but it is tempting to speculate that TAC-mediated growth is preferred to enhance calcium entry and support B-cell activation and differentiation. Accordingly, the proximity between the ER and cortical actin triggered by BCR activation could be attributed to ER-plasma membrane contacts established by STIM1. These contacts may involve several proteins, creating a mature subdomain that serves as a signaling platform for multiple processes (27). Moreover, ER tubule growth, known to promote inter-organelle interactions, could modulate lysosome and mitochondria fission, along with lipid transport and synthesis (18), which could further tune antigen extraction and processing.

Additionally, our research showed an increase of ER and KTN1 around the MTOC upon BCR activation. KTN1 is known to promote ER sheet formation in other cell types (20), however its function in B cells remains to be elucidated. Interestingly, nuclear repositioning in LPA-stimulated fibroblasts depends on the formation of ER sheets in the perinuclear region and requires proteins related to their formation and maintenance (28). Analogously, when B-cells encounter immobilized antigens, the nucleus reorientates its major groove towards the IS to organize antigen extraction machinery at the center and facilitate extraction and processing (29). Under this context, accumulation of the ER around the MTOC might enable the confinement of lysosomes as recently reported in other cell types (30), which upon local secretion can facilitate antigen extraction and internalization (5, 15).

BCR stimulation leads to an increase protein synthesis and upregulation of the secretory pathway (31) which is consistent with an increase in the formation of ER sheets, where active protein synthesis occurs, leading to higher ER sheet/tubule ratio in plasma cells (20). Intriguingly, the formation of ER exit sites-ERES is driven by the GTPase Rac1, a protein activated by the BCR signaling pathway (26). Thus, ER sheet formation is most likely initiated at early stages of B-cell activation, driven by antigen engagement by BCR.

B-cells encounter antigens in a soluble form, tethered to different cell types or surfaces, all of which display different stiffnesses. Our data show that this impacts how B cells reorganize the ER, where B-cells activated on softer surfaces did not uncouple the ER from microtubules and failed to completely redistribute the ER towards the IS. Interestingly, when analyzing the ER area at the synaptic interface of cells activated on 13kPa gels, despite actin spreading, the ER did not significantly increase its area. This suggests that ER extension at the IS might be activated by a distinct pathway than actin cytoskeleton spreading, and probably relies more on microtubule dynamics.

These differences in ER dynamics could be explained by enhanced BCR signaling, as stiffer substrates lead to the enhanced activation of critical signaling proteins like Syk and Tyr (10). Whether these variances in BCR activation in response to mechanical stimuli translate into increased antibody-secreting capacity of plasma cells has not been studied; however, we hypothesize that greater remodeling of the ER is intended to increase the secreting machinery of the cell for a better humoral immune response to antigens.

Our findings reveal that ER membrane remodeling occurs in B-cells at early stages of activation and propose that this is a critical step for B-cells to transform into antibody secreting plasma cells. Understanding the mechanisms that govern B-cell activation is essential to comprehend how pathological situations associated to changes in the physical properties of organs and extracellular matrix trigger the formation of autoreactive B-cells (9).

## Data Availability

The raw data supporting the conclusions of this article will be made available by the authors, without undue reservation.
